# Longitudinal analysis of brain structure using existence probability

**DOI:** 10.1002/brb3.1869

**Published:** 2020-10-09

**Authors:** Norihide Maikusa, Tadanori Fukami, Hiroshi Matsuda, The Japanese Alzheimer’s Disease Neuroimaging Initiative (J‐ADNI)

**Affiliations:** ^1^ Integrative Brain Imaging Center National Center of Neurology and Psychiatry Tokyo Japan; ^2^ Department of Informatics Faculty of Engineering Yamagata University Yamagata Japan; ^3^ Department of Radiology National Center of Neurology and Psychiatry Tokyo Japan

**Keywords:** Alzheimer's disease, longitudinal, MRI

## Abstract

**Introduction:**

We propose a method to evaluate quantitatively the longitudinal structural changes in brain atrophy to provide early detection of Alzheimer's disease (AD) and mild cognitive impairment (MCI).

**Methods:**

We used existence probabilities obtained by segmenting magnetic resonance (MR) images at two different time points into four regions: gray matter, white matter, cerebrospinal fluid, and background. This method was applied to T1‐weighted MR images of 110 participants with normal cognition (NL), 165 with MCI, and 82 with AD, obtained from the Japanese Alzheimer's Disease Neuroimaging Initiative database.

**Results:**

We obtained the coefficients of probability change (CPC) for each dataset. We found high area under the receiver operating characteristic curve (ROC) values (up to 0.908 of the difference of ROCs) for some CPC regions that are considered indicators of atrophy. Additionally, we attempted to establish a machine‐learning algorithm to classify participants as NL or AD. The maximum accuracy was 92.1% for NL‐AD classification and 81.2% for NL‐MCI classification using CPC values between images acquired at first and sixth months, respectively.

**Conclusion:**

These results showed that the proposed method is effective for the early detection of AD and MCI.

## Graphical abstract

This study established a novel, longitudinal method which uses machine‐learning algorithm to discriminate individuals with normal cognition (NL) from those with mild cognitive impairment (MCI) and Alzheimer's disease (AD). Our method achieves sufficient accuracy to discriminate NL from MCI and AD with minimally invasive scans obtained at only two time points (0 and 6 months).

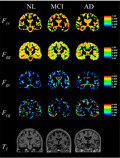

## 1 INTRODUCTION

Alzheimer's disease (AD) is the most common cause of dementia and typically manifests as memory impairment in the earliest clinical stage. Mild cognitive impairment (MCI), a less severe condition than AD, increases the risk of developing AD. Structural information of the brain provides novel insight to evaluate the progression of neurodegenerative disorders, such as MCI, AD, and from MCI to AD. Data can be analyzed with two approaches depending on whether the temporal or spatial axis is considered in cross‐sectional studies at an arbitrary time point and longitudinal studies at a fixed spatial location. Comparison between cognitively normal (NL) individuals and patients with AD is a common analysis method and helps explore the pathological condition underlying AD and MCI. However, the results of grouped data with this strategy may not necessarily be applied to individual cases. Longitudinal data analysis, including the characterization of longitudinal structural brain changes, is needed for superior prediction of the onset and treatment of AD.

Several established methods exist to analyze brain volume changes. The software package FreeSurfer longitudinal stream can estimate volume changes within more than 40 volumes of interest (VOIs) using the Bayes estimation and Markov random fields (Fischl et al., 2002, 2004). Tensor‐based morphometry compares the amount of deformation between two mutually registered images. This method performs global registration by affine transformation, with subsequent local registration by nonlinear transformation. The Jacobian map is obtained as the distribution of shrinkage and extension for each axis in the whole brain (Hua et al., 2010). Other methods focus on changes in the brain surface. Boundary shift integrals evaluate the degree of shrink by assessing changes in pixel values at the boundary between the cortical tissue and cerebrospinal fluid (CSF) (Freeborough & Fox, 1997; Leung et al., 2010, 2012). Using the distance between two corresponding voxels on a contour, structural image evaluation using the normalization of atrophy (SIENA) is another well‐known method to assess volume changes (Smith et al., 2002).

Numerous studies have used these longitudinal analyses to evaluate AD in magnetic resonance (MR) images; specifically, information regarding the hippocampus, such as its volume, shape, and structure, is often used to estimate the progress of AD (Ceyhan et al., 2011; Chan et al., 2001; Grundman et al., 2004; Mungas et al., 2002; Reuter et al., 2012; Tang et al., 2015).

Lillemark et al. (2014) investigated the relationship and proximity between brain regions to classify individuals into healthy control, MCI, and AD groups. Thompson et al. (2003) generated a map that visualized the rates of local gray matter loss over time. As aforementioned, most of these analyses have used the volume or shape of the whole or local brain regions. Reuter et al. (2012) developed a novel longitudinal image processing framework based on the FreeSurfer pipeline for automatic surface reconstruction and segmentation of brain MR images acquired at arbitrary time points.

This study proposes new indices for early detection of Alzheimer's disease and MCI by quantitative evaluation of longitudinal structural changes using corresponding changes in the brain tissue. Specifically, we used existence probabilities of the following three different tissues that can be easily obtained by segmentation in SPM8 (Statistical Parametric Mapping) (Ashburner & Friston, 2005): gray matter (GM), white matter (WM), and CSF. All other signals are considered background. The structural changes were considered changes in existence probabilities, and the temporal change in each brain tissue type was estimated from the above probabilities. The results were subsequently analyzed with the SPM software, a widely used tool to analyze brain MR images. This analysis enabled the acquisition of additional information with this method. Moreover, this method outlines both structural and tissue‐level changes. This report expounds on the method and its use to differentiate individuals with AD from those with NL, thereby demonstrating its classification efficacy.

## 2 MATERIALS AND METHODS

### 2.1 Subjects

We used T1‐weighted MR images from the Japanese Alzheimer's Disease Neuroimaging Initiative (J‐ADNI) dataset, provided by National Bioscience Database Center in Japan.

Data were acquired using 1.5 T MRI scanners (GE Healthcare, Siemens, and Philips). Scanning parameters are as follows: flip angle, 8°; inversion time, 1,000 ms; field of view, 240* × *240 mm^2^; slice thickness with no gap, 1.2 mm; repetition time, 2,400 ms for multicoil phased‐array head coil and 3,000 ms for birdcage coil for GE GNENESIS SIGNA, SIGNA EXCITE, SIGNA HDx/HDxt, Siemens Avanto, MEGNETOM, VISION, Sonata, Symphony, Symphony Vision, and Symphony Tim, 2,300 ms for multicoil phased‐array head coil for Philips Achieva; in plane resolution, 0.9375 × 0.9375 mm^2^, 1.0156 × 1.0156 mm^2^ or 1.25 × 1.25 mm^2^; acquisition plane, Sagittal; phase encoding direction, A/P.

All recruited volunteers were divided into the following three groups: (a) AD, (b) NL, (c) and MCI. All participants underwent examinations; these included MRI and cognitive assessments, such as the Mini‐Mental State Examination (MMSE) and the Clinical Dementia Rating (CDR). The J‐ADNI study was a longitudinal study for AD with MRI and cognitive assessments at 6‐month or 12‐month intervals for 2 or 3 years depending on the target group (for more details, refer to the J‐ADNI website). In this study, we used MRI performed at 0 and 6 months because if we can capture brain structural changes at short scanning intervals, it would be a more useful biomarker (Mubeen et al., 2017). Those who underwent an MRI scan with a different scanner at 0 and 6 months of age were excluded.

Since we found significant age difference between diagnostic groups on the original J‐ADNI dataset, age‐matched participants were chosen by stratified random sampling from the three groups. The MRI data provided by the J‐ADNI database were not isotropic. However, because this is a public database, this omission was outside of our control.

All data were collected after obtaining informed consent from participants and approval from the ethics committee of our hospital. Table 1 shows the detailed demographic information of the participants enrolled in this study.

**Table 1:** Participant demographics

	AD	MCI	NL	*p* value
*N*	82	165	110	
Age, mean (*SD*)	71.4 (6.70)	71.5 (6.37)	71.8 (6.28)	.891
Sex, M/F	49/60	79/83	32/49	.39
MMSE, mean (*SD*)	22.2 (1.70)	26.4 (1.71)	29.1 (1.26)	<.0001
Scanning interval days, mean (*SD*)	209 (18.9)	203 (12.5)	205 (13.9)	.0127
CDR, 0/0.5/1	0/54/28	0/165/0	110/0/0	

### 2.2 Image processing

All MR images were corrected for intensity inhomogeneity using the B1 correction algorithm (Narayana et al., 1988) and a nonparametric nonuniformity intensity normalization (N3) algorithm (Sled et al., 1998). Subsequently, phantom‐based distortion correction (Maikusa et al., 2013) was performed to normalize variations between MRI scanners.

**Figure 1:**
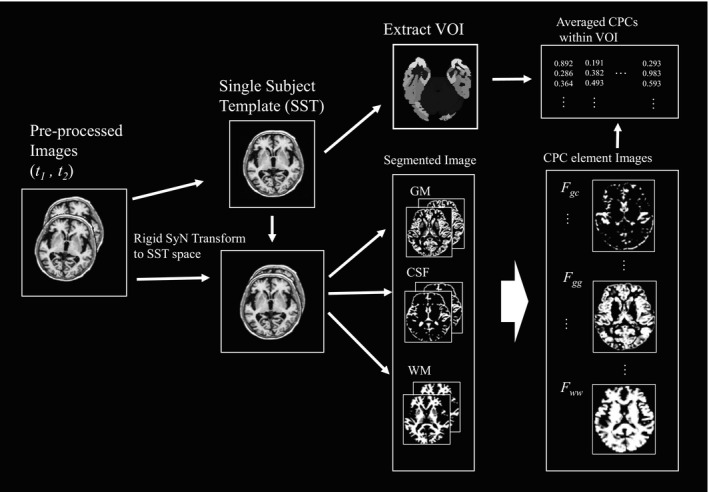
Flowchart to calculate CPC elements within the volume of interest from longitudinal brain structural images. CPC, coefficients of probability change

We constructed an automated pipeline to calculate the coefficient of probability change (CPC) elements within the VOIs as shown in Figure 1. This pipeline has four steps: VBM segmentation, creation of a single subject template (SST) and symmetrical registration, automatic extraction of the VOI, and calculation of the CPC elements.

#### 2.2.1 Create single subject template and symmetric registration

For unbiased longitudinal analysis, we first created an SST from individual MR images acquired at 0 and 6 months using the “antsMultivariateTemplateConstruction2.sh.” This script can create population‐specific or individual templates by coupling the intrinsic symmetric pairwise registration (Avants et al., 2010) with an optimized shape‐based sharpening/averaging template appearance. MR images acquired at two time points were subsequently registered to each SST.

#### 2.2.2 Segmentation

In the segmentation process, we used the VBM8 toolbox for image segmentation of SST‐registered MR images into three types of brain tissues (GM, WM, and CSF) and background. It is assumed that the histogram of image intensity follows a Gaussian mixture model. Accordingly, the existence possibilities of the three tissue types can be calculated for the image intensity at an arbitrary voxel, *p*(*T* | *I*), using the following Bayesian equation:

(1) $$p\left(T|I\right)=\frac{p(T)p(I|T)}{p(I)}$$

where *T* and *I* indicate the tissue type, including the background and voxel values, respectively. *p*(*T*) is the prior probability of the image and can be obtained from a standard template constructed using data from many participants. This template reflects the existence probability for each tissue, which is provided in SPM. *p*(*I* | *T*) is the likelihood, that is, the probability that the voxel has an intensity *I* when the tissue type is known. This value is easy to obtain because the intensity distribution for each tissue has already been calculated using the Gaussian mixture model. *p*(*I*) can also be obtained from the image histogram. The aforementioned four processing steps (realignment, registration, re‐slicing, and segmentation) were performed with SPM. We obtained the existence probabilities for the four tissues, including that for the background, at each voxel. These probabilities can be expressed as a vector with four elements.

#### 2.2.3 Coefficients with probability change

Here, we define the vector ***p*** ^(^ *^t^* ^)^ described in (2) at time *t* (with the first measurement as the reference time) for every voxel. The additional characters g, w, c, and b indicate GM, WM, CSF, and background, respectively. We denoted the vector at each measurement, excluding the reference time, as ***p*** ^(^ *^tt^* ^)^. We assumed a linear relationship between the two vectors, ***p*** ^(^ *^t^* ^)^ and ***p*** ^(^ *^tt^* ^)^, and describe the existence probabilities at an arbitrary voxel at two different time points, *t* and *t* ^′^, as [g^(^ *^t^* ^)^ w^(^ *^t^* ^)^ c^(^ *^t^* ^)^ b^(^ *^t^* ^)^]*^T^* and [g^(^ *^tt^* ^)^ w^(^ *^tt^* ^)^ c^(^ *^tt^* ^)^ b^(^ *^tt^* ^)^]*^T^*. That is, we assume that the existence probabilities at *t* ^′^ are defined as summation of the products of the probabilities at the first observation and the coefficients, which reflect the degree of change between the two observations. This relationship is expressed with the following equation:

(2) $$p\left(t^{t}\right)=F\cdot p^{\left(t\right)}$$

where

$$p^{\left(t^{\prime }\right)}=\left[\begin{array}{c} g^{\left(t^{\prime }\right)}\\ w^{\left(t^{\prime }\right)}\\ c^{\left(t^{\prime }\right)}\\ b^{\left(t^{\prime }\right)} \end{array}\right],\;p^{\left(t\right)}=\left[\begin{array}{c} g^{\left(t\right)}\\ w^{\left(t\right)}\\ c^{\left(t\right)}\\ b^{\left(t\right)} \end{array}\right],\;F=\left[\begin{array}{cccc} F_{\mathrm{gg}} & F_{\mathrm{wg}} & F_{\mathrm{cg}} & F_{\mathrm{bg}}\\ F_{\mathrm{gw}} & F_{\mathrm{ww}} & F_{\mathrm{cw}} & F_{\mathrm{bw}}\\ F_{\mathrm{gc}} & F_{\mathrm{wc}} & F_{\mathrm{cc}} & F_{\mathrm{bc}}\\ F_{\mathrm{gb}} & F_{\mathrm{wb}} & F_{\mathrm{cb}} & F_{\mathrm{bb}} \end{array}\right].$$

Here, the elements in matrix ***F*** in (2) indicate the degree of temporal change and are denoted as CPC herein. In this equation, 16 coefficients were required. However, only four equations are used for each voxel. Accordingly, with constraints, we used four voxels to obtain 16 coefficients.

Considering that 3* × *3 *× *3 voxels were used to obtain the coefficients, we used 108 equations. In addition, we specified the condition that CPC has positive values to ease clinical interpretation; therefore, the resulting non‐negative least‐squares problem was solved using the incorporated function in Matlab.

(3) $$\underset{F}{\arg\;\min}∥p^{\left(t^{\prime }\right)}‐F\cdot p^{\left(t\right)}∥,\;\text{where}\;\forall F_{\mathrm{ij}}\in F\geq 0$$

When the CPC matrix ***F*** is a unit matrix, the probabilities do not show structural changes. CPC in ***F*** can be obtained by constructing equations that describe changes in each tissue. As an example of change in GM using probabilities at the first measurement, *t*, and at another time point, *t* ^′^, we can obtain CPC values corresponding to the gray matter under the aforementioned constraints:

(4) $$\left[\begin{array}{c} g_{1}^{\left(t^{\prime }\right)}\\ g_{2}^{\left(t^{\prime }\right)}\\ g_{3}^{\left(t^{\prime }\right)}\\ \vdots \\ g_{27}^{\left(t^{\prime }\right)} \end{array}\right]=\left[\begin{array}{cccc} g_{1}^{\left(t\right)} & w_{1}^{\left(t\right)} & c_{1}^{\left(t\right)} & b_{1}^{\left(t\right)}\\ g_{2}^{\left(t\right)} & w_{2}^{\left(t\right)} & c_{2}^{\left(t\right)} & b_{2}^{\left(t\right)}\\ g_{3}^{\left(t\right)} & w_{3}^{\left(t\right)} & c_{3}^{\left(t\right)} & b_{3}^{\left(t\right)}\\ \vdots & \vdots & \vdots & \vdots \\ g_{27}^{\left(t\right)} & w_{27}^{\left(t\right)} & c_{27}^{\left(t\right)} & b_{27}^{\left(t\right)} \end{array}\right]\left[\begin{array}{c} F_{\mathrm{gg}}\\ F_{\mathrm{wg}}\\ F_{\mathrm{cg}}\\ F_{\mathrm{bg}} \end{array}\right]$$

The probability of the voxel being a part of the background was obtained by subtracting the total probabilities of GM, WM, and CSF from 1.0. Subsequently, probabilities in ***p*** ^(^ *^t^* ^)^ and ***p*** ^(^ *^tt^* ^)^ of less than 0.2 were neglected. Hence, if quantities of *g* ^(^ *^t^* ^)^, *w* ^(^ *^t^* ^)^, *c* ^(^ *^t^* ^)^, or *b* ^(^ *^t^* ^)^ for a given voxel that were less than 0.2 were set to 0, CPC becomes much larger when these possibilities are extremely small because it indicates the ratio of the probabilities at two different time points. Here, the maximal CPC is limited to 5.0 (1.0/ 0.2) by excluding probabilities less than 0.2. This approach specifically highlights the changes in tissues that comprise a sufficiently large proportion (more than 20%) of a voxel.

Accordingly, we set the value to 0 and adjusted the remaining components to satisfy the condition that the total existence probability is 1.0. CPCs greater or less than 1.0 indicate increase and decrease of probabilities, respectively. CPC located on a diagonal element in the matrix, *F_ii_*, indicates the change in existence probability between two different time points in a tissue. However, the other CPC, *F_ij_* (*i*/=*j*), shows the degree of contribution of probability of tissue *i* to that of tissue *j*. To compare the CPC and the direct longitudinal changes in anatomical probability, we performed a simple longitudinal analysis. We calculated the averaged rates of the posterior probability changes, that is, *gm*(*t* + 1)*/gm*(*t*), *wm*(*t* + 1)*/wm*(*t*), and *csf* (*t* + 1)*/csf* (*t*), within each VOIs at the two time points after affine registration to SST.

#### 2.2.4 Automatic extraction of the VOI

We analyzed each SST image with the joint label fusion method (Hongzhi et al., 2013). This method is effective to label a VOI automatically according to the multi‐atlas training set, Neuromorphometrics atlas (Neuromorphometrics Inc., 2016). This atlas features brain images of 30 participants from the J‐ADNI database, which have been manually labeled into 236 regions. These data are commercially available (Neuromorphometrics). Each VOI of the atlas is defined in Neuromorphometrics General Segmentation Protocol and by the BrainCOLOR Cortical Parcellation Protocol. After the extraction of VOIs, we calculated the average of each CPC element value within the extracted VOIs.

### 2.3 Machine‐learning classification

We constructed the classification models with machine learning and CPC elements according to the following two main steps:

1. We defined CPC elements to perform the classification. We focused on CPC components associated with brain atrophy. Specifically, in case of brain atrophy, the GM region will erode and the CSF region will dilate. Therefore, we employed *F_cc_*, *F_gg_*, *F_gc_*, and *F_cg_* as the input variables for machine‐learning classifications.
2. We then executed machine‐learning classification using three types of classifiers to detect AD: support vector machine (SVM), random forest (RF), and gradient boosting classifier (GBC). The CPC elements were averaged within the 236 regions. Machine‐learning classification permitted the integration of complementary information of CPC elements from different tissue types to potentially enable high‐performance classification. Furthermore, to avoid the dimensionality of the VOI approach, we used a principal component analysis (PCA) and thereby reduced the dimension of the features for machine learning. To tune the hyperparameters of each classifier, grid search was used to find the number of PCA components for all classifiers, including the optimal C (soft margin parameter) for linear SVM, C and gamma for rbf kernel SVM, the number of estimators and class weight for RF, and number of estimators and maximum depth for GBC. Other settings included the defaults of the scikit‐learn toolkit (version 0.19.1) running on the Python 3.6 platform. Classification accuracy was obtained by n‐fold cross‐validation, that is, data from 1/n participants were used for testing, whereas the others were used for training. In order to obtain stable results, we used 15‐fold cross‐validation to increase the number of training datasets as much as possible and sufficient number of test datasets. The classification results are validated using the mean values of accuracy (ACC), sensitivity (SEN), and specificity (SPE).

## 3 RESULTS

**Figure 2:**
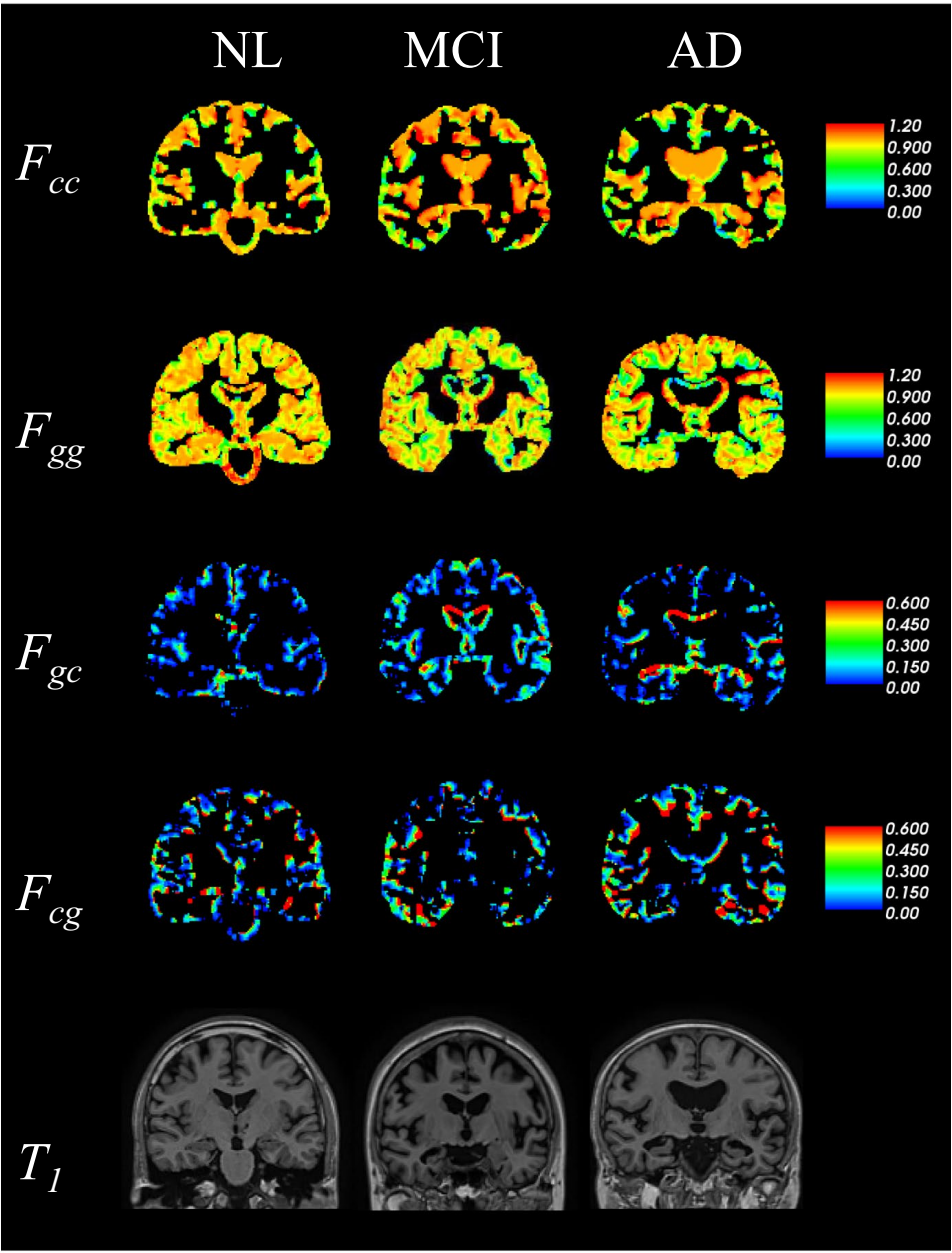
Representative images in NL (left column), MCI (middle column), and AD (right column) of each CPC element related to changes in GM and CSF (*F_cc_*, *F_gg_*, *F_gc_,* and *F_cg_*), and original T1‐weighted magnetic resonance image of SST. Color maps of image show CPC values. AD, Alzheimer's disease; CPC, coefficients of probability change; CSF, cerebrospinal fluid; GM, gray matter; MCI, mild cognitive impairment; NL, normal cognition

Figure 2 shows the spatial distributions of *F_gg_*, *F_cc_*, *F_gc_*, and *F_cg_,* and the target SST T1‐weighted image, which belongs to the NL and AD groups. The temporal changes in the representative indices using diagonal CPC (*F_cc_* and *F_gg_*) have values less than 1 in most voxels, and these values in participants with AD were lower locally than in those with NL. These elements represent the ratio of tissue perseverance; hence, a deviation of *F_gg_* and *F_cc_* from a value of 1 indicates the degree of change from GM and CSF to other regions, respectively. *F_gc_* indicates the probability of a tissue changing from gray matter to CSF, whereas *F_cg_* indicates the reverse. High values of *F_gc_* indicate GM atrophy. In Figure 2, high *F_gc_* values can be observed in participants with AD within the medial temporal areas, including the hippocampus, comparatively more than that in participants with NL. Moreover, we can see high *F_cg_* value boundary between GM and CSF in AD participant than NL.

Table 2 shows the top 20 regional AUCs from the ROC analysis between AD and NL, and MCI and NL groups using independent CPC elements. High AUC values in table correspond to the hippocampus, inferior lateral ventricle, and amygdala on ROC analysis between NL and AD groups. The trend between MCI and NL groups was similar to NL group versus.AD group. We also show results of ROC analysis using direct longitudinal changes in anatomical probability. The highest AUC value that could distinguish NL and AD was 0.864 within the right inferior lateral ventricle using CSF changes and 0.806 also within the right inferior lateral ventricle using CSF changes for NL and MCI. The values of AUC by CPC were higher than the direct longitudinal change in tissue probabilities.

**Table 2:** The top 20 areas under the receiver operating characteristic (ROC) curves of brain regions on ROC analysis of the differences between individuals with Alzheimer's disease and those with normal cognition determined using elements of coefficients of probability change

Rank	Element	Region	AUC
*NL versus AD*			
1	*F* *_cc_*	Left Hippocampus	0.908
2	*F* *_cc_*	Right Hippocampus	0.904
3	*F* *_gc_*	Right Hippocampus	0.883
4	*F* *_gc_*	Left Hippocampus	0.882
5	*F* *_cc_*	Right Inf Lat Vent	0.875
6	*F* *_gg_*	Left Inf Lat Vent	0.871
7	*F* *_gc_*	Left Inf Lat Vent	0.866
8	*F* *_gc_*	Right Inf Lat Vent	0.855
9	*F* *_cc_*	Left Amygdala	0.853
10	*F* *_gg_*	Left Amygdala	0.852
11	*F* *_cc_*	Left Inf Lat Vent	0.850
12	*F* *_gg_*	Right Inf Lat Vent	0.835
13	*F* *_gc_*	Left Amygdala	0.830
14	*F* *_cc_*	Right Amygdala	0.829
15	*F* *_cc_*	Left entorhinal area	0.821
16	*F* *_cc_*	Left PHG	0.813
17	*F* *_gc_*	Left entorhinal area	0.810
18	*F* *_gg_*	Right Amygdala Left	0.808
19	*F* *_gg_*	Thalamus Proper	0.802
20	*F* *_cc_*	Right PHG	0.786

Rank	Element	Region	AUC
*NL versus MCI*			
1	*F* *_cc_*	Right Hippocampus	0.863
2	*F* *_cc_*	Left Hippocampus	0.848
3	*F* *_cc_*	Right Inf Lat Vent	0.823
4	*F* *_cc_*	Left Inf Lat Vent	0.779
5	*F* *_gc_*	Right Hippocampus	0.778
6	*F* *_gc_*	Right Inf Lat Vent	0.770
7	*F* *_gc_*	Left Hippocampus	0.769
8	*F* *_gc_*	Left Inf Lat Vent	0.763
9	*F* *_gg_*	Left Amygdala	0.763
10	*F* *_gg_*	Right Inf Lat Vent	0.759
11	*F* *_cc_*	Right Hippocampus	0.757
12	*F* *_cc_*	Left Hippocampus	0.749
13	*F* *_gg_*	Right Inf Lat Vent	0.739
14	*F* *_gg_*	Left Inf Lat Vent	0.733
15	*F* *_gg_*	Right Hippocampus	0.731
16	*F* *_gg_*	Right Inf Lat Vent	0.730
17	*F* *_cc_*	Left Hippocampus	0.727
18	*F* *_cc_*	Left Inf Lat Vent	0.718
19	*F* *_gg_*	Left Amygdala	0.716
20	*F* *_gg_*	Right Inf Lat Vent	0.713

Note

The top row is a comparison of NL versus AD, and the bottom row is a comparison of NL versus MCI.

Abbreviations: PHG, Parahippocampal gyrus; Inf Lat Vent, inferior lateral ventricles.

The experimental results of individual structural changes and performance for differentiating between AD and MCI from NL by the machine‐learning algorithm are as follows. To assess the performance of the proposed CPC elements to detect and differentiate participants with AD from those with MCI, we used a machine‐learning classification with 15‐fold cross‐validation. Table 3 shows the results of machine‐learning classification of AD and MCI. With the combination of CPC elements (i.e., *F_gg_*, *F_cc_*, *F_gc_,* and *F_cg_*) and SVM classifier, the classification performances (accuracy, sensitivity, and specificity) to detect AD were 92.7%, 91.5%, and 93.6%, respectively. For each CPC element, the accuracy of *F_cc_*, *F_gg_*, and *F_gc_* was 90.1% 82.3%, and 84.4%, respectively.

**Table 3:** Best performances of the proposed method to detect AD and MCI using each CPC element (*F_gg_*, *F_cc_*, and *F_gc_*) alone and in combination

CPC Element	AD versus NL					NL versus MCI				
	Combined	*F_cc_*	*F_gg_*	*F_gc_*	*F_cg_*	Combined	*F_cc_*	*F_gg_*	*F_gc_*	*F_cg_*
Classifier	SVM	SVM	SVM	SVM	SVM	SVM	SVM	SVM	SVM	SVM
ACC (%)	**92.1**	91.1	82.1	84.7	80.5	**81.2**	79.7	79.0	74.9	79.7
SEN (%)	**88.9**	86.4	74.1	76.5	74.1	**84.0**	85.2	82.1	75.3	87.0
SPE (%)	**94.5**	94.5	88.1	90.8	85.3	**77.1**	71.6	74.3	74.3	68.8

Abbreviations: ACC, accuracy; SEN, sensitivity; SPE, specificity.

## 4 DISCUSSION

From the ROC analysis of the NL/AD and NL/MCI group classification within anatomical regions with *F_cc_*, *F_gc_*, and *F_gg_* elements, higher AUC values than direct longitudinal changes were found within the lateral temporal regions, which have been previously associated with AD. Hence, these elements can be considered more indicative of GM atrophy.

In Figure 2, participants with AD had lower *F_cc_* values than those with NL and MCI who had moderate *F_cc_* in the whole brain. This result is possibly indicative of the regions with CSF shifting to other regions due to the structural change induced by brain atrophy. In contrast, *F_cg_* has high values around the boundary between GM and CSF regions, where *F_cc_* showed a low value. This change from CSF to GM does not have biological significance. However, our method does not evaluate at the completely same voxel between two time points. If the brain is perfectly spherical and the atrophy is toward the center, then a change from CSF to GM cannot occur. However, at the boundary of the sulcus, for example, it is possible that atrophy causes a migration of gray matter to voxel where CSF is indicated at first time point. We believe that these parameters affect atrophy, but these complex changes in brain shape due to disease progression and atrophy are not clear, so we are not discussing it here, only assessing whether it is statistically different from healthy people. At the medial temporal and superior lateral ventricle, high *F_gc_* and low *F_gg_* were observed. Thus, it is considered that these CPC elements are indicative of GM atrophy.

A longitudinal machine‐learning approach consent from data obtained with fluorodeoxyglucose positron emission tomography (FDG PET) (over 12 months) was suggested by Gray et al. (2012), whereas another approach using MRI data collected at 0, 12, and 24 months since symptom onset was advanced by Farzan et al. (2015). The accuracies of FDG PET and MRI to distinguish AD from NL were 88.4% and 91.7%, respectively. A multimodal machine‐learning algorithm constructed from baseline data, including MRI, FDG PET, and CSF, was proposed by Gray et al. (2013); it achieved accuracies of 89.0% and 74.6% to distinguish AD from NL and MCI from NL, respectively. Zhang & Shen (2012) suggested an approach that incorporated the Apoe genotype into the multimodal model, including MRI, FDG PET, and CSF; it achieved an accuracy of 93.3% to distinguish AD from NL and 83.2% to distinguish MCI from NL. Westman et al. (2012) used MRI and CSF data to construct an orthogonal partial least‐squares to latent structures (OPLS) machine‐learning classifier that demonstrated accuracies of 91.8% and 77.6% to distinguish NL from AD and MCI, respectively (Westman et al., 2012). For data with only MRI and those with a single time point, Papakostas et al. (2015) developed a machine‐learning classifier using deformation‐ and voxel‐based morphometry. This model had an accuracy of 85.0% to discriminate AD from NL. Iman et al. constructed a classifier of histogram‐based, patient‐specific anatomical brain connectivity networks, which achieved accuracies of 84.2% and 70.4% to stratify NL from AD and MCI, respectively. Table 4 summarizes the performances of previously reported algorithms to distinguish NL from AD and MCI.

**Table 4:** Results of the stratification of individuals with NL from those with AD (top panel) and MCI (bottom panel)

Author	Data	NL/AD	Time point	Classifier	ACC (%)	SEN (%)	Spec (%)
Gray et al. (2012)	FDG	54/50	BL, 12M	SVM	88.4	83.2	93.6
Gray et al. (2013)	MRI, FDG, CSF, Apoe	35/37	BL	RF	89.0	87.9	90.0
Zhang and Shen (2012)	MRI, FDG, CSF, MRI	50/45	BL	SVM	93.3	N.A.	N.A.
Westman et al. (2012)	MRI	111/96	BL	OPLS	91.8	88.5	94.6
Papakostas et al. (2015)	MRI	49/49	BL	SVM	85.0	78.0	92.0
Farzan et al. (2015)	MRI	30/30	BL, 12M, 24M	SVM	91.7	90.0	93.3
Beheshti et al. (2017)	MRI	99/102	BL	SVM	84.2	88.8	79.0
Proposed	FDG	110/54	BL, 6M	SVM	92.1	88.9	94.5

Author	Data	NL/MCI	Time point	Classifier	ACC (%)	SEN (%)	Spec (%)
Gray et al. (2013)	MRI, FDG, CSF‐tau, Apoe	35/75	BL	RF	74.6	77.5	67.9
Zhang and Shen (2012)	MRI, FDG, CSF	50/91	BL	SVM	83.2	N.A.	N.A.
Westman et al. (2012)	MRI, CSF MRI	111/162	BL	OPLS	77.6	72.8	84.7
Beheshti et al. (2017)	MRI	99/98	BL	SVM	70.4	78.2	60.2
Proposed		165/110	BL, 6M	SVM	81.2	84.0	77.1

Note

These results are based on previously reported methods, as well as with the method proposed herein.

Abbreviations: OPLS, orthogonal partial least‐squares to latent structures; N.A., this metric is not available in the literature.

The multimodality classification frameworks consistently showed high performance; this result may be ascribed to the integration of complementary information contained in multimodality data, such as MRI, FDG PET, and CSF, into the machine‐learning algorithm. However, multimodality approaches require additional scanning costs. Moreover, PET scans have the risk of radiation exposure, whereas a lumbar puncture is required to sample CSF, which is an invasive procedure that exacerbates treatment burden. MRI‐based approaches alone are minimally invasive. However, the performance of classifiers based on cross‐sectional MRI data is relatively inefficient to distinguish AD/MCI/NL because of limited information of brain atrophy obtained through this method. Longitudinal approaches can provide additional information on brain, such as temporal progress of atrophy and therapeutic outcome. In fact, the longitudinal approaches of the present study, as well as those reported by Farzan et al., achieved superior accuracy relative to conventional cross‐sectional studies. The promptness and simplicity of the examinations required for longitudinal approach also reduce patient burden. With only two scans that are performed 6 months apart, our method demonstrates high accuracy as compared to other longitudinal approaches to distinguish individuals with NL and AD. 6 months is a very short time for follow‐up to identify longitudinal atrophy; however, Mubeen et al. (2017) demonstrated that short‐term, 6‐month, longitudinal assessments significantly enhanced the performance of AD prediction in comparison with the cross‐sectional model. Our method also demonstrates good accuracy to distinguish individuals with NL and MCI. The classification of NL and MCI is difficult because the effect size of changes in the brain between individuals with NL and MCI is small. Our method can distinguish individuals with MCI from those with NL with high sensitivity. Thus, our method will be a useful screening tool for individuals with MCI. Although the method used by Farzan et al. calculated whole‐brain atrophy rate, our approach divided the information related to longitudinal brain changes to different tissue types such as GM, WM, and CSF, into nine elements; thus, the noise and errors generated in the process of calculating brain atrophy and/or co‐registration between time points, for example, changes from GM to a background voxel that are sensitive to errors in detecting longitudinal changes, can be overcome by dividing the components using the CPC approach.

As shown in Table 4, all our results showed the best accuracy when using SVM. In general, RF and GBC are not suitable for cases where the sample size is not very large (Manuel et al., 2014). Our data had about 100 subjects in each group, which may explain why the SVM showed optimal results. Our method can detect brain preservation ratio and structural changes in the brain using only two brain MR images. The separated metrics with different characteristics, brain structural change, and preservation can be integrated by machine‐learning method. We believe that this is the reason why our method can achieve high accuracy with only MR images and short‐term longitudinal analysis.

Efficient retrieval of information with the longitudinal approach may account for this accuracy.

Moreover, our method can be expanded to broader applications with further development; for example, Fwg can be used to evaluate white matter hyperintensities (WMHs). WMH regions are also hypo‐intense on T1‐weighted MR images. So, areas of WMH are often misclassified by FSL and SPM routines that assess tissue probability using only signal intensity. To assess this, we would conduct future studies to assess whether WMH may be driving the differences in CPC elements, especially Fwg, compared with WMH volume by the SPM lesion segmentation tool. Moreover, the probability of tissue class from SPM in our method is based entirely on signal intensity. Several other groups have evaluated how change in signal intensity within tissues may predict Alzheimer's disease, such as boundary shift integral. We would also assess how the assessment of tissue probability technique compares with the assessment of change in T1 signal intensity for GM, WM, and CSF.

However, this study has some limitations. First, the time interval between two time scan was significantly different across diagnostic groups NL, AD and MCI due to disproportionately distributed data provided by the J‐ADNI database. Next, our method and other longitudinal measurement methods to calculate CPC elements are based on the assumption that registration of brain images from the two time points by rigid transformation can be performed almost completely. Therefore, our results and results of other longitudinal methods can include some measurement error bias derived from mis‐registration.

Our method also calculated other elements of CPCs that do not relate to brain tissue, that is, rather related to background. We believe that this coefficient related to background normally should be zero and can be used whether or not brain extraction worked identically during a longitudinal assessment. Therefore, we suggest that mis‐registration caused by unexpected head movements can be evaluated by CPC elements related to the background. We assessed averaged *F_bg_*, *F_bw_*, and *F_bc_* maps, and they were approximately zero; therefore, we think our results were likely not affected by head movements. But *F_bg_*, *F_bw_*, and *F_bc_* are insufficient for showing accurate registration of brain parenchyma (GM and WM). Consequently, we need new metrics to evaluate the registration success for a more accurate longitudinal assessment of brain.

## 5 CONCLUSIONS

We proposed a novel metric to detect brain changes using CPC values for GM, WM, and CSF. We evaluated the efficiency of the proposed metric obtained from baseline and 6‐month time points from the J‐ADNI database. We performed machine‐learning classification between NL versus AD and NL versus MCI. *F_gg_*, *F_cc_*, and *F_gc_* elements of CPC can reflect the previously characterized dynamics and neural manifestations of AD. Therefore, these elements can be used as surrogate biomarkers for computer‐aided diagnosis.

## CONFLICT OF INTEREST

The authors declare no conflicts of interest associated with this manuscript.

## AUTHOR CONTRIBUTION

N. Maikusa and T. Fukami developed the study concept and contributed to the study design. H. Matsuda involved in project administration and data collection. M. Maikusa performed the data analysis and interpretation. T. Fukami drafted the paper. All authors provided revisions and approved the final version of the paper for submission.

### Peer Review

The peer review history for this article is available at https://publons.com/publon/10.1002/brb3.1869.

## Data Availability

The datasets generated during the current study are available from the corresponding author upon reasonable request.
